# Radiographic and Clinical Comparison of Polyetheretherketone Versus 3D-Printed Titanium Cages in Lumbar Interbody Fusion—A Single Institution’s Experience

**DOI:** 10.3390/jcm14061813

**Published:** 2025-03-07

**Authors:** Diang Liu, Julie L. Chan, Art Eleanore, Kristin DeCost, Justin Luk, Lissette C. Neukam, Tasneem Zaihra Rizvi, Zhibang Lin, Zoher Ghogawala, Subu N. Magge, Andrew Y. Yew, Robert G. Whitmore

**Affiliations:** 1 Department of Neurosurgery, Lahey Hospital and Medical Center, Burlington, MA 01805, USA; diang.liu@tufts.edu (D.L.); lukj@bu.edu (J.L.); zoher.ghogawala@lahey.org (Z.G.); subu.n.magge@lahey.org (S.N.M.); andrew.y.yew@lahey.org (A.Y.Y.); robert.g.whitmore@lahey.org (R.G.W.); 2 Tufts University School of Medicine, Boston, MA 02111, USA; 3 Department of Neurosurgery, College of Medicine, University of Florida, Gainesville, FL 32608, USA

**Keywords:** lumbar fusion, PEEK, 3D-printed titanium, interbody cage

## Abstract

**Background/Objectives:** Spinal fusion surgery is an accepted form of management for select patients who suffer from degenerative lumbar disease. The need for cost-effective durable techniques is paramount as our population ages. This study compares the radiographic and clinical outcomes of PEEK and 3D-printed titanium interbody cages. **Methods:** This study compared two cohorts which underwent either PEEK or 3D-printed titanium (3DPT) interbody fusion at a single institution between 2013 and 2022. The PEEK cohort was a retrospective analysis of a prospectively collected registry. The 3DPT data were prospectively collected. The inclusion criteria were adults >18 years who underwent 1 or 2 level lumbar interbody fusion for degenerative spine disease with at least 6 months follow-up. Patient demographics, radiographs, and PROMs were collected. The cohorts were compared using ANOVA for continuous variables and Fisher’s exact test for categorical variables, with significance set to 0.05. **Results:** The final study included 91 patients, 49 PEEK and 42 3DPT. The 3DPT patients were older (*p* = 0.047) with increased incidence of hypertension (*p* < 0.001). The 3DPT patients had less bone morphogenetic protein (BMP) usage (80.9% vs. 54.8%; *p* = 0.012), but more cellular allograft (*p* < 0.001). Fusion rate was high for both cohorts, with PEEK at 95.9% and 3DPT at 97.6%. There was no significant difference in reoperation rate. Both the PEEK and 3DPT cohorts demonstrated an improvement in the Oswestry Disability Index (ODI) and EuroQol 5 Dimension (EQ-5D) at 1 and 2 years compared to preoperative baseline. More patients in the 3DPT group met the MCID for EQ-5D at 1 and 2 years compared to PEEK; however, this was not significant (*p* = 0.350; *p* = 1.000). **Conclusions**: The 3DPT interbody provided comparable if not superior fusion properties to the PEEK interbody given the decreased use of BMP. Both cohorts demonstrated similar improvements in ODI and EQ-5D compared to preoperative baseline. These results suggest that 3DPT cages may be a cost-effective alternative in spinal fusion. Further studies utilizing a larger population with higher follow-up rates are indicated to determine the economic and clinical benefits of 3DPT compared to PEEK cages in lumbar fusion surgery.

## 1. Introduction

Degenerative spinal disease is one of the most-treated pathologies in the United States (US). An estimated 266 million individuals worldwide suffer from symptomatic degenerative disc disease each year [1]. In the US alone, 16 million individuals are affected annually. Degenerative spinal disease may cause central canal stenosis, foraminal stenosis, and/or spondylolisthesis, leading to back pain, radiculopathy, and potentially irreversible neurologic deficit. While both non-operative and operative treatment options are available during the early stages of spinal disease, patients may ultimately require surgical intervention in the form of a spinal fusion.

Lumbar spinal fusion is an accepted treatment modality for spinal disease refractory to non-operative management. Of the estimated 1 million spinal fusions performed each year, many are reoperation procedures. Subsequently, there is a strong interest in improving the surgical durability and cost-effectiveness of spinal fusion surgery through new technology. Successful spine surgery can be assessed using multiple facets, including objective radiographic parameters as well as clinical outcome measures including patient-reported satisfaction scores. To improve patient outcomes following spinal surgeries, fusion techniques have evolved over the years with an emphasis on different approaches, biomechanical considerations, instrumentation technology, and biologic materials. Studies on the different approaches include comparisons of open vs. minimally invasive (MIS) and interbody placement techniques. Some studies suggest that MIS approaches may lead to better postoperative outcomes with lower reoperation rates; however, meta-analyses suggest there are no differences in long-term outcomes [2,3]. Similarly, there are debates regarding the optimal approach to interbody placement. However, a recent meta-analysis did not demonstrate any significant difference in clinical outcome or fusion rate when comparing transforaminal interbody fusion (TLIF), posterior lumbar interbody fusion (PLIF), and anterior lumbar interbody fusion (ALIF) [4]. However, the studies demonstrate that the circumferential approach and the use of an interbody implant increase the rate of successful arthrodesis [5]. As such, much of the contemporary research regarding spinal fusion has investigated the properties of interbody implants that provide improved postoperative outcomes.

Polyetheretherketone (PEEK) became widely used in spinal fusion procedures in the 1990s due to its elastic modulus, which is similar to that of cortical bone (4 GPa) [6]. While materials such as ceramic and carbon fiber have been proposed as substrates for interbody cages, titanium and PEEK remain the most extensively studied interbody materials in spine surgery. Titanium cages are of specific interest given their biologic properties, which promote spinal fusion. Titanium generates -TiO_2_ and promotes the stimulation of osteoblasts [7]. Further, titanium has a weight-to-strength ratio that withstands large compressive loads and, when mixed with carbon–fiber, it has a modulus of elasticity similar to cortical bone [8]. Manufacturing these cages through 3D-printing generates an appropriate porosity to allow for bony ingrowth through the cage, as well as providing a favorable modulus of elasticity [9]. Recent studies have demonstrated the non-inferiority and, in some cases, superiority regarding the fusion and subsidence rates of 3DPT cages over PEEK in lumbar interbody fusion. Kim et al. demonstrated that 3DPT cages not only had similar subsidence and fusion rates compared to PEEK, but the fusion quality promoted by the 3DPT cage was superior to PEEK at one year postoperatively [10]. Yang et al. compared fusion and subsidence rate in 150 patients who underwent PLIF and found similar subsidence rates and improved fusion rates with 3D-printed titanium (3DPT) compared to PEEK [11]. Other studies, such as that by Adl Amini et al., demonstrate that 3DPT may actually lead to decreased rates of subsidence at 12 months postoperatively compared to PEEK when utilized in standalone lateral interbody fusion [12]. 3DPT is particularly promising, as ovine models suggest that the porosity of the endplates and lattice structure of the 3DPT cages permits both bony ingrowth and mechanical stability during spinal fusion [13]. While studies regarding the benefits of 3DPT cages are encouraging, there are a paucity of studies which address durability, longer-term outcomes, and clinical correlation regarding 3DPT implants in lumbar interbody fusion. Here we report longer-term radiographic and clinical outcomes following PEEK and 3DPT lumbar interbody fusion.

## 2. Materials and Methods

### 2.1. Study Design

This is an institutional IRB-approved study (20213046) funded by DePuy Synthes (Johnson and Johnson, Raynham, MA, USA). This comparative study included two cohorts who underwent posterior lumbar interbody fusion for degenerative spondylotic disease at Beth Israel Lahey Hospital and Medical Center from 2013 to 2022. Patients had spinal fusion surgery at 1 or 2 levels via the PLIF or TLIF approach (open, mini-open, or MIS). The open approach consisted of a single midline incision, the mini-open approach included a small midline incision for interbody placement and bilateral percutaneous incisions for pedicle screw placement, and an MIS-tubular was performed via two bilateral paramedian incisions with a tubular retractor. The two cohorts were categorized into patients who received either PEEK or the 3DPT interbody device. The PEEK cohort was selected from the prospectively collected Lahey Clinic Lumbar Fusion registry. The 3DPT patients were enrolled in the study and data were collected prospectively. Inclusion criteria were adult patients 18 years or older, minimum 6 months radiographic follow-up (3DPT), and 1–2 years of follow-up (PEEK). Exclusion criteria included trauma, tumor, or osteoporosis defined by T-score < −2.5.

Demographics (age, sex, BMI) and comorbidities (diabetes mellitus, coronary artery disease, smoking status, hypertension, prior history of spine surgery) were collected from the lumbar fusion registry and/or electronic medical records. Patients’ presenting symptoms (back pain, leg pain, weakness, numbness/tingling), the number of levels fused, the lumbar level fused, the spondylolisthesis grade, the approach (PLIF vs. TLIF, open vs. mini-open vs. MIS-tubular), and the use of biologic bone extenders, estimated blood loss (EBL), and length of stay were also obtained from the medical records and/or fusion registry. Postoperative radiographs and patient-reported outcome measures (PROMs) including the Oswestry Disability Index (ODI) and EuroQol 5 Dimension (EQ-5D) scores were documented preoperatively and postoperatively at 3, 6, 12, and 24 months.

### 2.2. Radiographic Assessment

Postoperative radiographs (XR or CT) were evaluated for bony fusion and subsidence across the instrumented level(s) by two blinded neurosurgeons. Successful fusion was based on the Bridwell Interbody Fusion Grading System where Grades I and II were considered fused [14,15] (Table 1). Subsidence was graded according to the Marchi classification system, which determines the level of subsidence using the amount of disc space collapse (Grade 0: 0–24%; Grade I: 25–49%; Grade II: 50–74%; Grade III: 75–100% collapse of the disc space). For the purposes of this study, Grade 0 was considered as no subsidence [16]. If discordance occurred between the reviewers, a third neurosurgeon reviewed the radiographic films for the final determination.

### 2.3. Patient-Reported Outcomes Measures

PROMs were obtained through ODI or EQ-5D questionnaires delivered in the outpatient setting at 3, 6, 12, and 24 months. For those who did not present to the office setting, the appropriate forms were mailed to the patient and then returned to the study coordinator. Change in ODI and EQ-5D from preoperative baseline were calculated at 1 and 2 years postoperatively. In addition, minimal clinically important difference (MCID) was applied to assess patients’ change in ODI and EQ-5D relative to a standardized metric. An MCID of 14.9 was utilized for ODI based on a study by Parker et al., which determined the MCID for patients who underwent TLIF for spondylolisthesis [17]. For EQ-5D, an MCID of 0.19 was selected based on a study by Burgstaller et al. which calculated the MCID for patients who underwent spine surgery for lumbar spinal stenosis [18].

### 2.4. Statistical Analysis

Baseline demographic and surgical variables were compared between PEEK and 3DPT using univariate analysis. ANOVA was used for continuous variables and Fisher’s exact test for comparing categorical variables. All tests were two-tailed, unless otherwise mentioned, with the significance level set to 0.05. The multivariate models were adjusted for any statistically and/or clinically significant demographic or clinical variables (i.e., age and hypertension) for change in baseline PROMs at 1- and 2-years postoperatively. We fitted mixed models with random intercept to account for the correlation among PROMs (ODI and EQ-5D) within patients at 1- and 2-year time points. The models were adjusted for time, age, and hypertension given the significant difference between the PEEK and 3DPT cohorts (Multiple imputation was performed to alleviate the poor follow-up rates. A sensitivity analysis comparing imputed models with complete case analyses did not indicate any significant difference in the model in terms of parameter estimates or inference. All analyses were completed in RStudio, R version 4.3.3 [19].

## 3. Results

### 3.1. Patient Demographics

The PEEK cohort included 49 patients; 32 underwent PLIF and 17 underwent TLIF. Forty-two patients were enrolled in the 3DPT cohort: twenty PLIF and twenty-two TLIF. Most patients underwent open procedures (PEEK 55.1%, 27/49; 3DPT 76.2%, 32/42), a subset underwent the mini-open procedure (PEEK 44.9% 22/49; 3DPT 23.8%, 10/42), and some underwent the MIS-tubular procedure (PEEK 8.2% 4/49, 3DPT 9.5%, 4/42). Among patients who received PEEK interbody devices, the mean age was 59.67 years, of which 53% were female (26/49). Patients who received 3DPT interbody devices had a mean age of 64.26 years, with 36% being female (15/42). For the majority of the demographics and baseline surgical variables, there was no significant difference between the two cohorts. A borderline significant difference in age was observed between the PEEK and 3DPT cohorts (PEEK 59.67 vs. 3DPT 64.26; *p* = 0.047), and the 3DPT patients had a higher incidence of hypertension (3DPT 66.7%, 28/42 vs. PEEK 6.1%, 3/49; *p* < 0.001) (Table 2).

### 3.2. Perioperative Analyses

Patients in the PEEK and 3DPT cohorts did not differ in terms of presenting symptoms (back pain, leg pain, numbness/tingling, weakness), spondylolisthesis grade, number of surgical levels, or interbody fusion level at L4–5 or L5–S1. However, a statistically significant difference was observed between the PEEK and 3DPT patients regarding L3–4 fusion level, mini-open vs. open approach, EBL, and cellular allograft/bone morphogenetic protein (BMP) use. The 3DPT patients had more surgeries performed at L3–4 (3DPT 26.2%, 11/42 vs. PEEK 6.1%, 3/49; *p* = 0.010), and more cases were open surgeries (3DPT 76.2%, 32/42 vs. PEEK 55.1% 27/49; *p* = 0.048). There was a significant difference in estimated blood loss (EBL), with the patients with titanium cages experiencing roughly half as much blood loss (3DPT 227.38 ± 153.40 vs. PEEK 525.00 ± 394.84; *p* < 0.001). Despite the difference in EBL, the cohorts did not differ in length of stay. PEEK patients stayed a mean of 4.35 ± 2.99 days and the 3DPT patients stayed for a mean of 4.26 ± 2.91 days (*p* = 0.883). Patients with titanium cages had less BMP usage (3DPT 54.8%, 23/42 vs. PEEK 80.9%, 38/47; *p* = 0.012) and more cellular allograft placement (3DPT 45.2%, 19/42 vs. PEEK 12.8%, 6/47; *p* < 0.001). There was no difference in reoperation rate (PEEK 6.1%, 3/49 vs. 3DPT 4.8%, 2/42; *p* = 1.00). Only five patients returned to the OR over 2 years—three PEEK patients (3/49, 6.1%) and two 3DPT patients (2/42, 4.8%). One PEEK patient returned to the OR within the first postoperative year for pedicle screw revision. In year 2, two patients in each cohort required revision surgery for adjacent segment disease.

### 3.3. Radiographic Analysis

**Figure 1:**
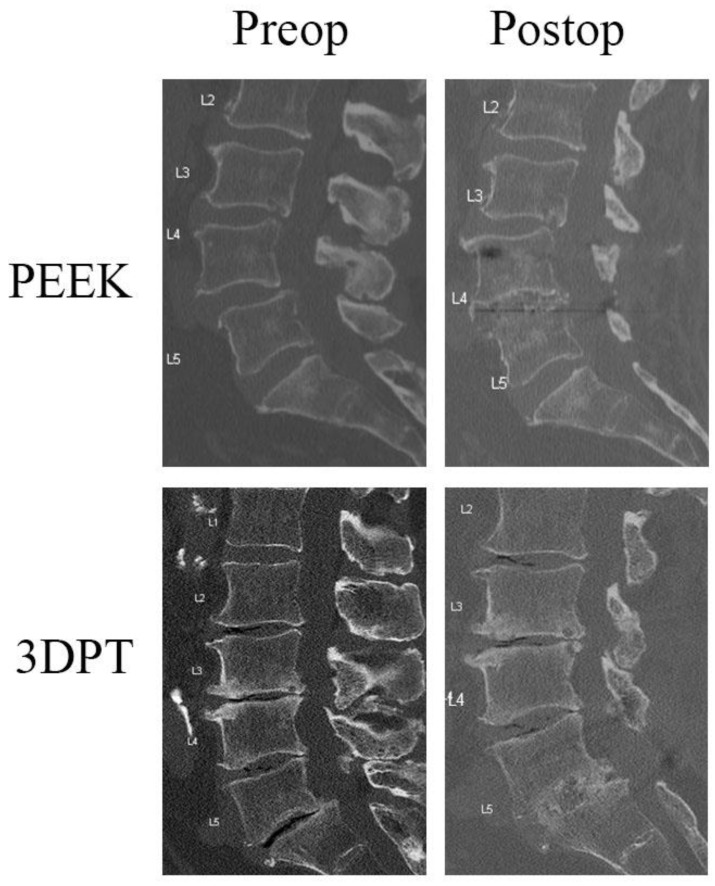
Representative preoperative and postoperative images of patients who underwent lumbar interbody fusion with either PEEK or 3DPT cage. Patient 1’s preoperative sagittal CT demonstrating L4–5 grade 1 spondylolisthesis and postoperative CT of L4–5 PLIF with PEEK implant at 3 years postoperatively. Patient 2’s preoperative sagittal CT with L5-S1 grade 1 spondylolisthesis, and 2-year postoperative CT of L5-S1 PLIF with 3DPT implant.

Almost all patients in both groups demonstrated fusion at 1 year postoperatively, with a fusion rate of 95.9% (47/49) for PEEK and 97.6% (41/42) for 3DPT (*p* = 1.000). Of the patients that failed to fuse at 1 year in the PEEK cohort, one patient underwent open PLIF, and the other underwent MIS-tubular TLIF. In the 3DPT cohort, the one patient that failed to fuse underwent open TLIF. Subgroup analyses of PLIF/TLIF and open/mini-open/MIS-tubular did not demonstrate any significant differences in fusion postoperatively (PLIF/TLIF *p* = 0.131; open vs. mini-open *p* = 0.073; open vs. MIS-tubular *p* = 0.700). Figure 1 contains representative images of patients’ preoperative and postoperative radiographic images with a PEEK or 3DPT cage. The Marchi classification system was utilized to evaluate for subsidence in postoperative radiographs. All patients included in this study demonstrated 0–24% disc collapse at 1 year postoperatively. This was classified as Grade 0 and these patients were determined to have no subsidence.

### 3.4. Patient Reported Outcomes

ODI and EQ-5D were compared at 1 and 2 years postoperatively. The PEEK cohort (*n* = 49) had a PROM follow-up rate of 86% and 53% at one and two years, respectively. The 3DPT cohort (*n* = 42) had a follow-up rate of 33% at one year and 45% at two years. At one year postoperatively, there was no difference in change from preoperative baseline ODI or EQ-5D between the PEEK and 3DPT cohorts. Specifically, PEEK patients had a mean change in ODI of 24.45 ± 17.03 and a mean change in EQ-5D of −0.22 ± 0.22, while 3DPT patients had a mean change in ODI of 18.22 ± 14.32 and a mean change in EQ-5D of −0.22 ± 0.17 (*p* = 0.224 and *p* = 0.927, respectively). Similarly, there was no significant difference in PROMs at two years postoperatively. PEEK patients had a mean change in ODI of 25.69 ± 13.39 and a mean change in EQ-5D of −0.22 ± 0.24, compared to a mean change in ODI of 19.37 ± 19.78 and a mean change in EQ-5D of −0.19 ± 0.24 in the 3DPT interbody device cohort at 2 years postoperatively (*p* = 0.208 and *p* = 0.658, respectively) (Table 3).

When MCID was applied as a standardized metric at one year postoperatively, 73.8% and 52.4% of PEEK patients met the MCID for ODI (14.9) and EQ-5D (0.19), respectively. At two years postoperatively, 84.6% and 53.8% of patients achieved MCID for ODI and EQ-5D, respectively. For the 3DPT patients at one year postoperatively, 64.3% and 71.4% of patients achieved MCID for ODI and EQ-5D, respectively. At two years, 68.4% and 57.9% of the 3DPT patients achieved MCID for ODI and EQ-5D, respectively. There was no statistical significance in the achievement of MCID between the PEEK and 3DPT cohorts for either ODI or EQ-5D at 1 or 2 years postoperatively. Further subgroup analysis did not demonstrate any differences in PROMs when stratified by approach (PLIF/TLIF, open/mini-open/MIS-tubular). Finally, a multivariate analysis was performed and adjusted for any baseline characteristics that were statistically and/or clinically different. Mixed models with random intercept were fitted to account for within-patient correlation between PROMs (ODI or EQ-5D) scores at the 1- and 2-year time points. The models were adjusted for time, age, and hypertension given the significant difference between the PEEK and 3DPT cohorts. A subgroup analysis of the interbody approach and open/mini-open/MIS did not show significance between the two cohorts for all outcomes. Further, a multiple imputation and sensitivity analysis comparing the imputed models with complete case analyses did not indicate any significant difference in the model in terms of parameter estimates or inference. Following these more rigorous analyses, no differences in ODI or EQ-5D were noted at 1 or 2 years between the PEEK and 3DPT cohorts.

## 4. Discussion

Spinal fusion surgery still has significant room for growth regarding postoperative radiographic and clinical outcomes. As such, the interest in developing an improved technology to optimize spine fusion outcomes continues to evolve. While there is no consensus regarding the ideal interbody for optimal clinical outcomes following lumbar fusion surgery, the literature suggests that titanium interbody devices may have superior fusion rates compared to PEEK. However, improved fusion rates may come at the expense of a higher risk for subsidence [20,21]. To mitigate the potential for cage subsidence while retaining the bioactive properties of titanium, the development of porous designs has reduced the elastic modulus of titanium closer to that of cortical bone as well as permitting osseointegration [9,11]. A recent study by Toop et al. compared the subsidence rate between solid titanium interbody devices and 3DPT interbody devices, which showed a significantly lower risk of subsidence in the 3DPT cohort (5.5% vs. 24.4% at 12 months) [14]. Similarly, longer-term studies by Yang et al. confirm that 3DPT subsidence in PLIF has a similar rate to that of PEEK at 2 years postoperatively, confirming its non-inferiority [11].

Our study aligns with the prior literature, which demonstrates that 3DPT interbodies provide a comparable fusion success to that of PEEK cages. The radiologic analyses in our study confirmed that 3DPT interbody devices have an excellent fusion rate with no observed cases of subsidence. The lack of subsidence in both the PEEK and 3DPT cohorts supports that the risk of subsidence is reduced with porous 3D-printed versions of titanium compared to solid implants [14]. This study also demonstrates that 3DPT cages are capable of achieving a reliable rate of solid bony fusion; however, there was no significant difference between the 3DPT and PEEK fusion rates. The lack of significance between the two cohorts may be due to the high baseline fusion rate of PEEK in our patient cohort. Other studies in the literature that compared titanium and PEEK cages had much lower rates of fusion for PEEK interbodies in PLIF and TLIF (50–60%) [15,22]—nearly half the fusion rate in our study. While fusion and subsidence are important radiographic and mechanical factors to consider when assessing successful interbody integration, one aspect of interbody fusion that our study did not assess is interbody cage migration. Owing to 3D printing and the inherent properties of titanium, Ti cages are thought to promote better osseointegration, thereby reducing the potential for cage migration, as seen with the PEEK interbodies in lumbar fusion [23]. Additional studies specifically evaluating cage migration should be considered. If 3D-printed titanium cages do indeed permit better integration, they may reduce the need for supportive biologics such as bone-extenders.

In our study, the 3DPT group had lower rates of BMP usage, and an increase in the use of cellular allografts. While this potentially represents a chronological shift in surgeons’ practice, and is not the primary endpoint evaluated, this suggests that porous titanium implants may mitigate the need for certain biologics, such as BMP. While the 3DPT cohort still utilized bone-extenders, the potential to use less BMP has implications from both economic and clinical safety profiles. Numerous studies have specifically shown BMP to directly increase the cost of surgery, with some authors documenting the mean direct cost of BMP to be USD 10,444 ± 4607, and as high as USD 17,271 in spinal fusion surgery [24,25,26]. In addition to the cost considerations, BMP has been linked to postoperative complications such as heterotopic ossification and osteolysis, which may lead to repeat surgical interventions [27,28]. Further, there are some studies that suggest that BMP may be linked to increased rates of cancer [29,30]. Together, these results suggest that interbody implants such as 3DPT, which achieve spinal fusion without the use of costly bone-extenders, may be advantageous compared to the widely used PEEK implants from both economic and long-term clinical standpoints.

Complementing the fusion success rate of our patients, both the PEEK and 3DPT cohorts did well postoperatively, with low reoperation rates at two years postoperatively. The PEEK cohort cumulative reoperation rate was 6.1% at two years, which is slightly less than the 7% rate demonstrated by a meta-analysis by Peng et al., but more similar to the 5.7% obtained in a study of a propensity-matched cohort analysis by Hikata et al. [31]. At two years, our 3DPT cohorts demonstrated a slightly decreased reoperation rate (4.8%) compared to PEEK, similar to the rate pf 5.2% observed following TLIF in other studies [32]. Interestingly, larger series evaluating lumbar fusion suggest that reoperation for adjacent segment disease (ASD) following lumbar surgery increases with time and may be as high as 9% at 4.7 years postoperatively [33]. The two-year reoperation rates for both cohorts were only 4.8%for ASD. While our reoperation rates appear to be in line with the current literature, longer-term data points may shed light on the impact of fusion quality in the development of ASD between the PEEK and 3DPT cohorts. As such, future studies which include both radiographic and clinical outcomes over longer time periods will likely provide valuable insight into the nuances of the 3DPT cages that facilitate durable spinal fusion.

While many studies in the literature have evaluated visual analog scale (VAS) scores with respect to spinal fusion outcomes, few studies have evaluated ODI or EQ-5D, specifically comparing PEEK and 3DPT interbody use. A meta-analysis by Li et al. reported on five studies which utilized ODI as a measurement of functional recovery following Ti-coated PEEK and PEEK interbodies. Overall, this meta-analysis demonstrated that Ti-PEEK patients had improved ODI scores early on, at <6 months postoperatively, but no difference was noted in the longer term [34]. Similarly, a single-surgeon study of 93 patients who underwent spinal fusion with silicate-substituted calcium phosphate-packed 3DPT cages demonstrated a significant improvement in physical functioning for both the TLIF and lateral lumbar interbody fusion (LLIF) approaches [35]. Together, these studies suggest that patients who undergo spinal fusion with 3DPT cages may benefit clinically from solid fusion without subsidence in the longer term. However, the clinical outcomes of 3DPT cages compared to PEEK remain unknown. While there was no statistical significance across the two groups regarding changes in ODI and EQ-5D, our study supports that clinical outcomes following interbody fusion are comparable between PEEK and 3DPT, with a notable clinical improvement in ODI compared to the baseline at 1 and 2 years postoperatively.

This study has several limitations. Most notably, this is a single-institution study that incorporated variable operative techniques, including both PLIF and TLIF as well as minimally invasive approaches, introducing significant variability. Despite the potential for this heterogeneity to introduce bias or confounders, our subgroup analysis did not demonstrate any statistical difference between MIS/mini-open/open or TLIF/PLIF among the cohorts regarding fusion rate or clinical outcomes. These clinical outcomes are consistent with the 5-year follow up of MIS and open TLIF patients in a multicenter prospective study from the Quality Outcomes Database registry [36].

Our study may also have introduced bias with the study design by comparing a historical cohort collected from a fusion registry with a prospective cohort. Due to the nature of this study design, using data collected at different time periods, the rate of follow-up was poor at 2 years, with 53% and 45% follow-up for PEEK and 3DPT, respectively. This poor follow-up rate may skew the analysis of the longer-term patient-reported outcomes between PEEK and 3DPT cages. These data suggest that additional studies with a head-to-head comparison may be required to further understand the long-term clinical effects of the implant material, particularly as surgical trends tend to evolve over time.

The application of additional analyses, including multiple imputations, attempted to account for the poor follow-up rate; however, the trends remained the same, likely due the already small sample size. Given that patients in both cohorts demonstrated an improvement in ODI over time—while not significant—this does suggest that an improved follow-up may provide a more detailed understanding of any potential differences in outcomes between the two cohorts. While the number of patients achieving ODI MCID was not statistically significant in either cohort at 1 or 2 years postoperatively, the improvement in outcomes may still be clinically meaningful. Studies which evaluate the utility of MCID demonstrate that the selection of MCID can vary significantly depending on the calculation technique [37]. In a study by Parker et al. [37], the authors found that the calculation of MCID for adjacent segment disease ranged from 6.8 to 16.9 for ODI, and from 0.27 to 0.54 for EQ-5D. These and similar studies demonstrate that MCID is variable and may be specific to particular patient populations; therefore, utilizing the MCID from other patient cohorts may not always capture clinically relevant changes in PROMs.

Specific to our study, our PROM data may not only be affected by the variability in MCID thresholds, but our follow-up rate of 50% likely further affected our results, which failed to accurately determine the number of patients who achieved MCID. While additional analyses, such as multiple imputation and multivariate analyses, were utilized to strengthen the findings of the study, these more rigorous analyses found no statistical difference in PROMs between the PEEK and 3DtPT cohorts. The lack of statistical difference between these two cohorts may be influenced by the low follow-up rates and also the high rates of fusion and lack of subsidence, leading to a decreased need for reoperation and subsequently equivocal clinical outcomes. Notably, EQ-5D scores had less variability than ODI scores, potentially emphasizing the impact that additional clinical, non-surgical factors may affect certain PROMs. These differences underscore the importance of utilizing multiple PROMs when assessing clinical outcomes following spinal fusion surgery [38].

Overall, 3D-printed porous titanium implants demonstrate comparable rates of fusion, subsidence, reoperation rates, and postoperative clinical outcomes to PEEK cages in lumbar interbody fusion. Further studies comparing these interbody implants should be considered as the innate properties of 3DPT cages may provide advantages that reduce the economic and long-term clinical burden of spine surgery.

## Figures and Tables

**Table 1:** Bridwell Interbody Fusion Grading System.

Grade	Description
I	Fusion with remodeling and trabeculae
II	Graft intact without full incorporation or remodeling, but no lucency present.
III	Graft intact, potential lucency above and below implant
IV	No fusion, implant collapse and/or resorption

**Table 2:** Demographic characteristics and surgical variables.

	PEEK (N = 49)	3DPT (N = 42)	*p*-Value
	Mean (SD)	Mean (SD)	
Age (years)	59.67 (11.07)	64.26 (10.60)	0.047 * ^(1)^
BMI (kg/m^2^)	30.68 (7.07)	32.23 (5.75)	0.259 ^(1)^
Sex	**No (%)**	**No (%)**	
Male	23 (46.9%)	27 (64.3%)	0.139 ^(2)^
Female	26 (53.1%)	15 (35.7%)	
Comorbidity			
Diabetes Mellitus	6 (12.2%)	5 (11.9%)	1.000 ^(2)^
Coronary Artery Disease	2 (4.1%)	2 (4.8%)	1.000 ^(2)^
Hypertension	3 (6.1%)	28 (66.7%)	<0.001 * ^(2)^
Smoking Status (+nicotine level)	3 (6.1%)	2 (4.8%)	1.000 ^(2)^
History of Spine Surgery	10 (20.4%)	6 (14.3%)	0.583 ^(2)^
Presenting Symptom			
Back Pain	46 (93.9%)	35 (83.3%)	0.178 ^(2)^
Leg Pain	43 (87.8%)	36 (85.7%)	1.000 ^(2)^
Numbness/Tingling	8 (16.3%)	5 (11.9%)	0.765 ^(2)^
Weakness	6 (12.2%)	3 (7.1%)	0.498 ^(2)^
Spondylolisthesis			
Grade 1	40 (81.6%)	33 (78.5%)	0.70 ^(2)^
Grade 2	3 (6.1%)	5 (11.9%)	0.33 ^(2)^
Number of Surgical Levels			
1	35 (71.4%)	31 (73.8%)	0.819 ^(2)^
2	14 (28.6%)	11 (26.2%)	
Interbody Fusion Level			
L3–4	3 (6.1%)	11 (26.2%)	0.010 * ^(2)^
L4–5	39 (79.6%)	26 (61.9%)	0.102 ^(2)^
L5–S1	21 (42.9%)	16 (38.1%)	0.674 ^(2)^
Interbody Placement			
PLIF	32 (65.3%)	20 (47.6%)	0.096 ^(2)^
TLIF	17 (34.7%)	22 (52.4%)	
Open	27 (55.1%)	32 (76.2%)	
Mini-open	22 (44.9%)	10 (23.8%)	0.048 * ^(2)^
MIS-tubular	4 (8.2%)	4 (9.5%)	1.000 ^(2)^
Bone Morphogenetic Protein	38 (80.9%)	23 (54.8%)	0.012 * ^(2)^
Cellular Allograft	6 (12.8%)	19 (45.2%)	<0.001 * ^(2)^
	**Mean (SD)**	**Mean (SD)**	
Estimated Blood Loss (mL)	525.00 (394.84)	227.38 (153.40)	<0.001 * ^(1)^
Length of Stay (days)	4.35 (2.99)	4.26 (2.91)	0.883 ^(1)^

* α = 0.05; ^(1)^ Linear model ANOVA; ^(2)^ Fisher’s Exact Test for count data.

**Table 3:** Postoperative radiographic and clinical outcomes.

	PEEK (N = 49)	3DPT (N = 42)	*p*-Value
Return to OR	No (%)	No (%)	
1-year	1 (2.0%)	0	1.000 ^(1)^
2-year	2 (4.1%)	2 (4.8%)	1.000 ^(1)^
Cumulative 2-year	3 (6.1%)	2 (4.8%)	1.000 ^(1)^
Fusion	47 (95.9%)	41 (97.6%)	1.000 ^(1)^
ODI MCID 1-year (14.9)	31 (73.8%)	9 (64.3%)	0.511 ^(1)^
ODI MCID 2-year (14.9)	22 (84.6%)	13 (68.4%)	0.281 ^(1)^
EQ-5D MCID 1-year (0.19)	22 (52.4%)	10 (71.4%)	0.350 ^(1)^
EQ-5D MCID 2-year (0.19)	14 (53.8%)	11 (57.9%)	1.000 ^(1)^
	Mean (SD)	Mean (SD)	
ODI Change 1-year	24.45 (17.03)	18.22 (14.32)	0.224 ^(2)^
ODI Change 2-year	25.69 (13.39)	19.37 (19.78)	0.208 ^(2)^
EQ-5D Change 1-year	−0.22 (0.22)	−0.22 (0.17)	0.927 ^(2)^
EQ-5D Change 2-year	−0.22 (0.24)	−0.19 (0.24)	0.658 ^(2)^

^(1)^ Fisher’s Exact Test for count data; ^(2)^ linear model ANOVA.

## Data Availability

The datasets presented in this article are not readily available due to technical and time limitations. Requests for access should be directed to Lahey Hospital and Medical Center.

## Abbreviations

The following abbreviations are used in this manuscript:

ALIF	Anterior lumbar interbody fusion
BMP	Bone morphogenetic protein
EQ-5D	EuroQol-5D
GPa	Gigapascal
LLIF	Lateral lumbar interbody fusion
ODI	Oswestry Disability Index
PEEK	Polyetheretherketone
PLIF	Posterior lumbar interbody fusion
PROM	Patient reported outcome measure
TLIF	Transforaminal lumbar interbody fusion
US	United States
VAS	Visual analog scale
3DPT	3D-printed titanium
